# Adaption and validation of the nasal obstruction symptom evaluation scale in German language (D-NOSE)

**DOI:** 10.1186/s12955-018-1004-x

**Published:** 2018-09-04

**Authors:** Christoph Spiekermann, Eleftherios Savvas, Claudia Rudack, Markus Stenner

**Affiliations:** 1 Department of Otorhinolaryngology, Head and Neck Surgery, University Hospital Münster, Kardinal-von-Galen-Ring 10, 48149 Münster, Germany; 2 Institute of Immunology, University Hospital Münster, Münster, Germany

**Keywords:** Cross-cultural adaption, Validation study, Outcome, Quality of life, Nasal obstruction, Rhinoplasty, Allergy

## Abstract

**Background:**

Questionnaires have proven their worth in detecting changes in quality of life after medical interventions. The Nasal Obstruction Symptom Evaluation scale (NOSE) is a reliable and valid tool to identify restrictions of quality of life in patients with nasal problems. The aim of this prospective study was the validation of the German version of the NOSE scale (D-NOSE).

**Methods:**

Adaption of the NOSE in German language was performed by forward and backward translation process. Patients undergoing functional septorhinoplasty were asked to complete the D-NOSE preoperatively, one, three or twelve months after surgery. Healthy volunteers served as controls. Reliability, validity and responsiveness of the D-NOSE were determined.

**Results:**

The D-NOSE showed a good internal consistency as well as good inter-item, item-total correlation and a satisfactory test-retest reliability. The convincing validity of the adapted NOSE scale was approved by good construct validity and an excellent discriminant validity. Furthermore, a high sensitivity to identify clinical changes due to an intervention indicates a good responsiveness of the D-NOSE.

**Conclusions:**

The adapted German version of the NOSE questionnaire (D-NOSE) is an appropriate and validated tool to assess the influence of nasal obstruction in quality of life in German speaking patients.

## Background

Nasal obstruction is often a common complaint in patients seeking otolaryngological consultation and can also be a pivotal motive in patients undergoing functional septorhinoplasty. Hence, success of septorhinoplasty also depends on the patient’s subjective satisfaction with the functional result. Although objective measurements are essential for the clinician to properly document and follow-up on complaints of nasal obstruction, subjective questionnaires are becoming an established and valuable instrument to assess patient-reported outcome in nasal surgery. Brief questionnaires have proven their worth in quickly evaluating surgical outcomes and are appropriate tools for internal quality management [1–4].

A validated and reliable questionnaire is the “Nasal Obstruction Symptom Evaluation (NOSE)” Score, developed by Stewart et al., consisting of five questions concerning subjective assessment of the nasal obstruction within the past month. It is a brief disease-specific instrument (Table 1). Each question can be answered using a 5-point Likert scale from “0” (not a problem) up to “4” (severe problems with breathing). After addition of all item values and multiplying the raw score with 5, severity of the patient’s complaints can be scaled to range from 0 to 100. A score of 0 indicates no obstructive nasal problems and a score of 100 implies severe problems. It was described to have an internal consistency with a Cronbach-α of 0.785 and an adequate test-retest reliability with a coefficient of γ = 0.702. Furthermore, excellent discrimination between patients and control group was shown to be possible (*p* < 0.001) [5].

**Table 1:** Original version of the NOSE scale with adapted translations in German language (italic)

Over the *past 1 month*, how much of a problem were the following conditions for you? *Inwieweit waren die folgenden Zustände in den letzten 4 Wochen* *ein Problem für Sie?*					
	not a problem *kein Problem*	very mild problem *sehr geringes Problem*	moderate problem *mäßiges Problem*	fairly bad problem *recht großes Problem*	severe problem *schweres Problem*
Nasal congestion or stuffiness *Verstopfung der Nase*	□	□	□	□	□
Nasal blockage or obstruction *Engegefühl in der Nase*	□	□	□	□	□
Trouble breathing through my nose *Probleme durch die Nase zu atmen*	□	□	□	□	□
Trouble sleeping *Probleme zu schlafen*	□	□	□	□	□
Unable to get enough air through my nose during exercise or exertion *Unfähigkeit beim Sport genug Luft durch die Nase zu bekommen*	□	□	□	□	□

Cross-cultural adaptions and validations are necessary for international comparison of studies and methodologies. Hence, the NOSE questionnaire has been already successfully adapted to French, Greek, Chinese, Italian, Portuguese, Slovenian, Dutch, Spanic, Arabic and recently to Turkish language with a validity and reliability similar to the original version [6–15]. Because of its great impact it was our aim to translate and validate the NOSE scale in the German language.

## Methods

### Design

This prospective cross-cultural adaption and validation study was performed between May 2013 and November 2016 at the Department of Otorhinolaryngology, Head and Neck Surgery, University Hospital Münster.

### Patients and data acquisition

Patients undergoing functional septorhinoplasty were included in this study. The patients were asked to complete the translated NOSE questionnaire during outpatient consultation preoperatively, four weeks, three months and up to one year after surgery. Furthermore, the patients had to assess their difficulties with breathing through the nose in general. Patients with isolated septoplasty or concomitant procedures, especially sinus surgery were excluded. Healthy volunteers without nasal problems served as controls. The study was approved by the institutional review board (Ethik Kommission der Ärztekammer Westfalen-Lippe und der Westfälischen Wilhelms-Universität, 2016–418-f-S) and informed consent was obtained from all subjects.

### Questionnaire

The adaption of the “Nasal Obstruction Symptom Evaluation (NOSE) Scale” from English to German (D-NOSE) included a forward and backward translation process as described previously [16]. Two independent, bilingual German-native speakers with medical background translated the original English version into German. A consensus version was developed by discussion and revision of the translated versions by the authors. Backward translation of the consensus version was performed independently by two English native speakers with and without medical background as well as an English speaking professional who were all familiar with cultural and linguistic nuances of the original and translated language [17]. Hence, adequacy of the translated version was proven by comparison of the original with the backward-translated versions. (Table 1).

### Reliability

The reliability of a test can be defined by its internal consistency and its test-retest reliability. Cronbach’s α values were determined for the internal consistency. Internal consistency was considered to be fair (0.7 ≤ α ≤ 0.79), good (0.8 ≤ α ≤ 0.89) or excellent (α ≥ 0.9). Furthermore, two-way mixed intraclass correlation coefficients (ICC) were calculated with split-half-method for further confirmation of the internal consistency [18]. Corrected item-total and inter-item correlations were determined by Spearman correlation (0.2 < r_sp_ ≤ 0.5: low correlation, 0.5 < r_sp_ ≤ 0.8: good correlation, 0.8 < r_sp_ ≤ 1.0: excellent correlation).

Test-retest reliability was measured with the split-half method. Therefore, patients were assigned to compared groups by odd-even method. A sufficient test-retest reliability was assumed for *p* > 0.05.

### Validity

The validity consists of the construct and the discriminant validity. For determination of the construct validity we used an adapted version of the second question (ROE-Q2) of the “Rhinoplasty Outcome Evaluation” questionnaire [19]. This question is concerned about the possibility to breathe through the nose in general and can be answered by using a five point Likert scale with a range from 0 (severe problems) to 4 (no problems). Spearman correlations (see above) between the NOSE sum score and the ROE-Q2 were determined to describe the content validity.

38 healthy volunteers were asked to complete the translated NOSE questionnaire as well as the ROE-Q2. Student t -test was performed for determination of the discriminant validity.

### Responsiveness

The responsiveness was measured by comparison of the preoperative and postoperative NOSE sum scores with Wilcoxon rank test.

### Statistical analysis

Correlations (r_sp_ or ICC) are described with the 95% confidential interval (95%CI). Student t-test was performed for parametric independent results and paired t-test for parametric linked variables. Mann-Whitney-U test was used for independent non-parametric variables. Linked non-parametric variables were analyzed by Wilcoxon rank sum test. Results with *p* ≥ 0.05 were considered not to be significant. Statistical evaluation was performed with IBM® SPSS® Statistics 24.

## Results

### Patients

With a return rate of approximately 57% of the questionnaires, 207 patients who underwent functional septorhinoplasty could be included. Due to the data acquisition at different time points, 335 completed NOSE questionnaires were available for analyses. With 111 males and 96 females, the male to female ratio was 1:0.86. Male patients underwent rhinoplasty with a median age of 25 years (range from 15 to 63) and female patients with a median age of 26 years (range from 16 to 70 years). The healthy controls (*n* = 38) had a median age of 30 years (range from 21 to 58)**.** The participants were able to complete the questionnaire within 2 min in median (range 1–4 min) without any problems of understanding.

### Reliability

High internal consistency of the translated NOSE score was proven by Cronbach’s α = 0.87 preoperatively and α = 0.90 one, three and twelve months after surgery. Intraclass correlation coefficients confirmed the high internal consistency at each time point (ICC = 0.87 (95%CI: 0.82; 0.90) preoperatively, ICC = 0.90 (95%CI: 0.80; 0.96) postoperatively, *p* < 0.001). The inter-item and item-total correlations are illustrated in Table 2. Split-half method revealed no significant differences of the mean NOSE scores between the compared groups so that a sufficient test-retest reliability is assumable (58.1 ± 24.0 vs. 58.5 ± 22.9 (mean ± SD), *p* = 0.934).

**Table 2:** Inter-item and item-total correlations of the NOSE questionnaire (values are Spearman correlation coefficients = r_sp_, ^*^p < 0.001)

Item	NOSE-Q1	NOSE-Q2	NOSE-Q3	NOSE-Q4	NOSE-Q5
NOSE-Q2	0.514^*^				
NOSE-Q3	0.670^*^	0.642^*^			
NOSE-Q4	0.431^*^	0.501^*^	0.571^*^		
NOSE-Q5	0.515^*^	0.548^*^	0.638^*^	0.587^*^	
NOSE score	0.733^*^	0.784^*^	0.857^*^	0.793^*^	0.809^*^

*Abbreviations*: *Q* Question

### Validity

**Fig. 1:**
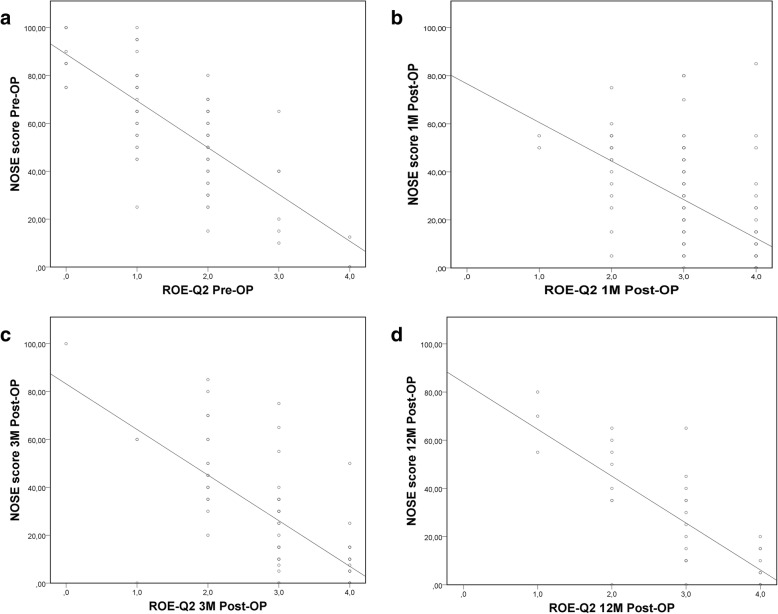
Correlations of the NOSE score with the Question ROE-Q2 preoperatively (**a**), one (**b**), three (**c**) and twelve months (**d**) after surgery. Abbreviations: NOSE = Nasal Obstruction Symptom Evaluation, ROE-Q2 = second question of the Rhinoplasty Outcome Evaluation questionnaire, M = month/s

Good negative correlations of the NOSE score with the ROE-Q2 could be observed at every time point of acquisition - indicating a good construct validity of the adapted NOSE questionnaire (preoperatively: r_s*p*_ = − 0.73 (95%CI: -4.6; − 3.22), one month: r_s*p*_ = − 0.62 (− 4.14; − 2.29), three months: r_s*p*_ = − 0.75 (− 4.50; − 3.12), and twelve months: r_s*p*_ = − 0.74 (− 4.94; − 2.74), *p* < 0.001) after surgery (Fig. 1).

Comparison of the patients (preoperatively) with the healthy controls revealed significant differences of the NOSE scores (58.3 ± 23.4 preoperatively vs 14.6 ± 16.3, *p* < 0.001).

### Responsiveness

Wilcoxon rank sum test was performed to identify significant differences between pre- and postoperative NOSE scores. Preoperatively the patients had a median NOSE score of 60 (range from 45 to 75). One month or 12 months after surgery the NOSE score was diminished to a median score of 20 (5–45) (*p* < 0.001). Three month after the procedure the median NOSE score was about 15 (5–35) (*p* < 0.001). These results indicate a high sensitivity of the adapted NOSE questionnaire to identify clinical changes due to an intervention.

## Discussion

Questionnaires play a pivotal role in the assessment and evaluation of surgical outcomes. For an appropriate use in clinical practice, the questionnaires should be clear and comprehensible for the patients and their validity and reliability has to be proven. Every translation and adaption to another language carries the risk of a loss of information, validity or reliability. Hence, the aim of the present study was to validate and determine the reliability of the German version of the NOSE.

The internal consistency describes the correlation between the different items of the questionnaire [20]. With a Cronbach α = 0.87 a good internal consistency was observed. This is in congruence with the consistency of the French NOSE (α = 0.86) but is higher than in the original version (α = 0.79) [5, 6]. Analysis of the test-retest reliability is necessary to determine the reproducibility of the instrument. No differences between the half-split groups indicate a good test-retest reliability of the D-NOSE which has already been proven for NOSE in other languages [13].

The good negative correlation of the NOSE sum score with the second question of the ROE impressively demonstrates the construct validity of the D-NOSE. A German translated version of the ROE (ROE-D) was recently validated but was not available at the beginning of this study [21]. However, the translated versions of the second question of the ROE show just a slight difference. The discriminatory validity is an important feature of a questionnaire in clinical practice and describes the capacity to differentiate between affected and not affected/healthy groups [22]. Significant differences between the NOSE sum score of the patients and the healthy controls reveal an excellent discriminatory validity to identify patients with nasal obstruction.

Especially in assessment of surgical outcome, the applied instrument should provide the sensitivity to detect changes due to an intervention. This feature of a questionnaire or an instrument is called responsiveness [22]. A significant decrease of NOSE sum scores one, three or twelve months after the procedure compared to the preoperative score could be observed and represents a good responsiveness of the adapted D-NOSE.

Overall, the German version of the NOSE questionnaire showed a good reliability, a good validity and a high sensitivity to changes. Unfortunately, there was a low study participation leading to a return rate of 57%. Patients who gave their consent to study enrollment, however, showed a very good acceptance of the NOSE questionnaire, as it has been proven in several other studies [2, 5, 9, 15].

The NOSE instrument, however, was validated for groups and not individuals. Consequently, it can be a useful tool to compare patients’ pre and post therapeutical status or to evaluate different therapy strategies according to the outcome. But assessment with the NOSE scale allows no predictions concerning the outcome of individual patients [5]. The NOSE scale was developed to assess the subjective perception of nasal obstruction. Combining and comparing D-NOSE scores with objective measurements preoperatively should have all the relevant information necessary to choose the appropriate therapy and hence, to improve the outcome and the quality of life of patients with symptoms of nasal obstruction.

## Conclusions

The adapted German version of the NOSE questionnaire (D-NOSE) is an appropriate tool to assess the influence of nasal obstruction on quality of life in German speaking patients. Validity, reliability and responsiveness of the D-NOSE are in concordance with the original version and verify the successful adaption process.

## Abbreviations

95%CI: 95% confidential interval
D-NOSE: German version of the NOSE questionnaire
ICC: Intraclass correlation coefficients
NOSE: Nasal Obstruction Symptom Evaluation scale
ROE: Rhinoplasty Outcome Evaluation
ROE-Q2: Second question of the ROE
SD: Standard Deviation

## References

[1] Kahraman E, Cil Y, Incesulu A (2016). The effect of nasal obstruction after different nasal surgeries using acoustic Rhinometry and nasal obstruction symptom evaluation scale. World J Plast Surg.

[2] Kahveci OK, Miman MC, Yucel A, Yucedag F, Okur E, Altuntas A (2012). The efficiency of Nose Obstruction Symptom Evaluation (NOSE) scale on patients with nasal septal deviation. Auris Nasus Larynx.

[3] Gandomi B, Bayat A, Kazemei T (2010). Outcomes of septoplasty in young adults: the nasal obstruction septoplasty effectiveness study. Am J Otolaryngol.

[4] Lavinsky-Wolff M, Dolci JE, Camargo HL, Manzini M, Petersen S, Romanczuk S, Pizzoni R, Polanczyk CA (2013). Vertical dome division: a quality-of-life outcome study. Otolaryngol Head Neck Surg.

[5] Stewart MG, Witsell DL, Smith TL, Weaver EM, Yueh B, Hannley MT (2004). Development and validation of the nasal obstruction symptom evaluation (NOSE) scale. Otolaryngol Head Neck Surg.

[6] Marro M, Mondina M, Stoll D, de Gabory L (2011). French validation of the NOSE and RhinoQOL questionnaires in the management of nasal obstruction. Otolaryngol Head Neck Surg.

[7] Lachanas VA, Tsiouvaka S, Tsea M, Hajiioannou JK, Skoulakis CE (2014). Validation of the nasal obstruction symptom evaluation (NOSE) scale for Greek patients. Otolaryngol Head Neck Surg.

[8] Dong D, Zhao Y, Stewart MG, Sun L, Cheng H, Wang J, Li W (2014). Development of the Chinese nasal obstruction symptom evaluation (NOSE) questionnaire. Zhonghua Er Bi Yan Hou Tou Jing Wai Ke Za Zhi.

[9] Mozzanica F, Urbani E, Atac M, Scotta G, Luciano K, Bulgheroni C, De Cristofaro V, Gera R, Schindler A, Ottaviani F (2013). Reliability and validity of the Italian nose obstruction symptom evaluation (I-NOSE) scale. Eur Arch Otorhinolaryngol.

[10] Bezerra TF, Padua FG, Pilan RR, Stewart MG, Voegels RL (2011). Cross-cultural adaptation and validation of a quality of life questionnaire: the nasal obstruction symptom evaluation questionnaire. Rhinology.

[11] Larrosa F, Roura J, Dura MJ, Guirao M, Alberti A, Alobid I (2015). Adaptation and validation of the Spanish version of the nasal obstruction symptom evaluation (NOSE) scale. Rhinology.

[12] Urbancic J, Soklic Kosak T, Jenko K, Bozanic Urbancic N, Hudoklin P, Delakorda M, Juvanec A, Zupancic Urbancic K, Vadnjal J, Gluvajic D (2016). Cross-cultural adaptation and validation of nasal obstruction symptom evaluation questionnaire in Slovenian language. Zdr Varst.

[13] van Zijl FVWJ, Timman R, Datema FR (2017). Adaptation and validation of the Dutch version of the nasal obstruction symptom evaluation (NOSE) scale. Eur Arch Otorhinolaryngol.

[14] Amer MA, Kabbash IA, Younes A, Elzayat S, Tomoum MO (2017). Validation and cross-cultural adaptation of the arabic version of the nasal obstruction symptom evaluation scale. Laryngoscope.

[15] Onerci Celebi O, Araz Server E, Yigit O, Longur ES (2018). Adaptation and validation of the Turkish version of the nasal obstruction symptom evaluation scale. Int Forum Allergy Rhinol.

[16] Spiekermann C, Rudack C, Stenner M. Reliability and validity of the German version of the Utrecht questionnaire for outcome assessment in aesthetic rhinoplasty (D-OAR). Eur Arch Otorhinolaryngol. 2017;274(11):3893–8. 10.1007/s00405-017-4706-5 28815302

[17] Sousa VD, Rojjanasrirat W (2011). Translation, adaptation and validation of instruments or scales for use in cross-cultural health care research: a clear and user-friendly guideline. J Eval Clin Pract.

[18] Shrout PE, Fleiss JL (1979). Intraclass correlations: uses in assessing rater reliability. Psychol Bull.

[19] Alsarraf R, Larrabee WF, Jr AS, Murakami CS, Johnson CM (2001). Measuring cosmetic facial plastic surgery outcomes: a pilot study. Arch Facial Plast Surg.

[20] LJ C (1947). Test reliability; its meaning and determination. Psychometrika.

[21] Bulut OC, Plinkert PK, Wallner F, Baumann I (2016). Quality of life in functional rhinoplasty: rhinoplasty outcomes evaluation German version (ROE-D). Eur Arch Otorhinolaryngol.

[22] Guyatt GH, Kirshner B, Jaeschke R (1992). Measuring health status: what are the necessary measurement properties?. J Clin Epidemiol.

